# Optimization of workflow processes for sustainable paternal involvement: case study of an academic “daddy surgeon” in Japan

**DOI:** 10.1007/s00595-024-02959-y

**Published:** 2024-12-07

**Authors:** Nobuhiko Kanaya, Shinji Kuroda, Yoshitaka Kondo, Yuko Takehara, Yoshihiko Kakiuchi, Hitoshi Minagi, Masaki Sakamoto, Shunsuke Kagawa, Hitomi Kataoka, Toshiyoshi Fujiwara

**Affiliations:** 1 Department of Gastroenterological Surgery, Dentistry and Pharmaceutical Sciences, Okayama University Graduate School of Medicine, 2-5-1 Shikata-cho, Kita-ku, Okayama, 700-8558 Japan; 2 Department of Surgery, Okayama Saiseikai General Hospital, 2-25, Kokutai-cho, Kita-ku, Okayama, 700-8511 Japan; 3 Integrated Clinical Education Center, Kyoto University Hospital, 54 Kawahara-choShogoin, Sakyo-ku, Kyoto, 606-8397 Japan

**Keywords:** Optimization of workflow processes, Sustainable paternity participation, “Daddy surgeon”

## Abstract

Work–life balance is often discussed in Japan. Yet surgeons find it challenging to take paternity leave because of their demanding surgical duties and a strong sense of responsibility. One Japanese male surgeon had his first paternity experience as a research fellow in the US. When he returned to Japan, he resumed his surgical training and started a research project to become an academic surgeon. When he and his wife were expecting their second child, they discussed his paternity participation before the delivery and decided on a sustainable paternity participation plan. By coordinating his responsibilities with his co-workers, he limited his attendance at work to daytime hours only for 1 month to manage paternity duties. This adjustment did not affect the number of main and assistant operations conducted that month and effective optimization of workflow processes decreased the extra workload for other team members. His experience suggests that the optimization of workflow processes can enhance personal life, including paternity participation. (150/150).

In Japanese society, the importance of paternity leave for men is gaining recognition, with the acquisition rate reaching 17.13% in 2022, according to the Basic Survey on Equal Employment by the Ministry of Health, Labor and Welfare. Japan’s government encourages fathers to take paternity leave to promote equal opportunities for men and women.

A medical practitioner’s workload involves the management of patients’ health, emergency duties, skill training, and on-going learning of new evidence. Gastroenterological surgeons in particular face intense training demands over several years to achieve professional standards in Japan. Recently, the importance of work–life balance has been raised within the surgical community. In fact, at the recent Annual Congress of the Japanese Surgical Association, it was suggested that surgeons should have the opportunity to take paternity leave based on individual lifestyles. In Japan, 90% of surgeons are men [1], and the majority do not take sufficient paternity leave because of their intense surgical demands, including emergency cases, and the limited cover available, as well as a lack of paternity leave experience among peers.

A 38-year-old male surgeon, who had taken paternity leave when he worked as a research fellow in the US after general surgical training, recognized the importance of balance between his life and career plan through diversity. After he completed his 4-year research fellowship in the US, he returned to Japan to accept a clinical fellow position in a team of five male gastric and colorectal surgeons, treating benign diseases such as inflammatory bowel disease as well as gastric and colorectal cancers. In his first year, his wife became pregnant with their second child and they proactively discussed paternity participation before delivery. They decided on his specific childcare responsibilities for a month after her delivery, including picking up and dropping off their first child up at his childcare, bathing their children, and other aspects of paternity participation at night. He did not take long paternity leave because of financial constraints, which in turn influenced their decision to prioritize active childcare involvement over extended paternity leave. He discussed his plans with his clinical team and co-workers including senior colleagues who had had paternity experience and understood its importance and difficulties. His team members were all were supportive of his paternity participation, and he planned a working schedule for the 1 month after his wife’s delivery (Fig. 1). He took 3 days of paternity leave from the date of her discharge from hospital after the delivery. Thereafter, he started working the daytime hours of 8:30 am–5:15 pm.

**Fig.1:**
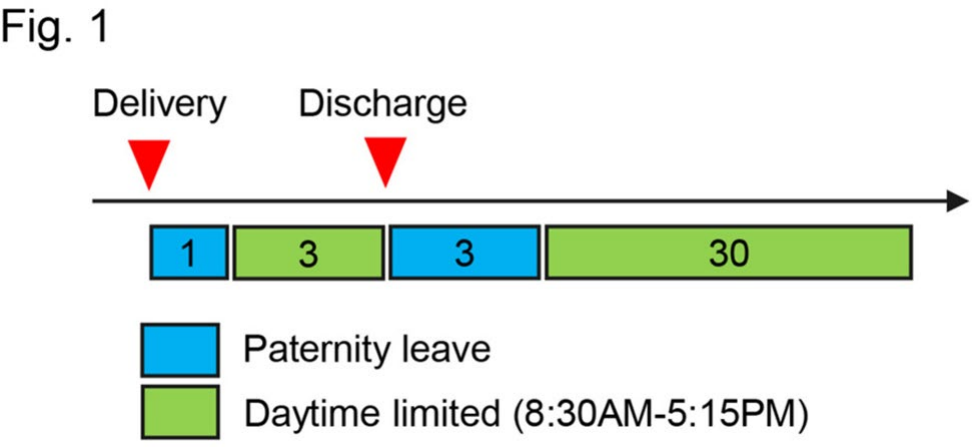
Working shift pattern of the “daddy surgeon” during his paternity participation. Scheme showing his working schedule during his paternity participation. Each number represents the number of days

To support the success of his paternity participation, his team made some changes to their working routines. Mainly, they covered the task shifts of the surgeons’ work to reduce wasted time in their team. For example, each member managed informed consent from the patients and their family, patient care after surgery, and specimen arrangements. In his limited free time during the daytime, he and a resident prepared the patient presentation slides for pre-/post-operative evaluation and wrote official documents. Despite the daytime shifts, he did not change the number of main or assistant operations although he could not participate in the patients’ care during the night. Furthermore, he applied for three research grant proposals even though he could not spend enough time on his research experiments. To assess the impact on his colleagues, the team compared the extra time worked during his paternity participation month to that during the previous month. Interestingly, all co-workers reported no increase in extra working time from the previous month (Fig. 2). He is currently still participating in paternity duties such as dropping his child off at childcare before work, which is made possible by the team’s work efficiency. His wife is happy with the outcome of his participation because their children are growing up well with the shared parenting responsibilities.

**Fig.2:**
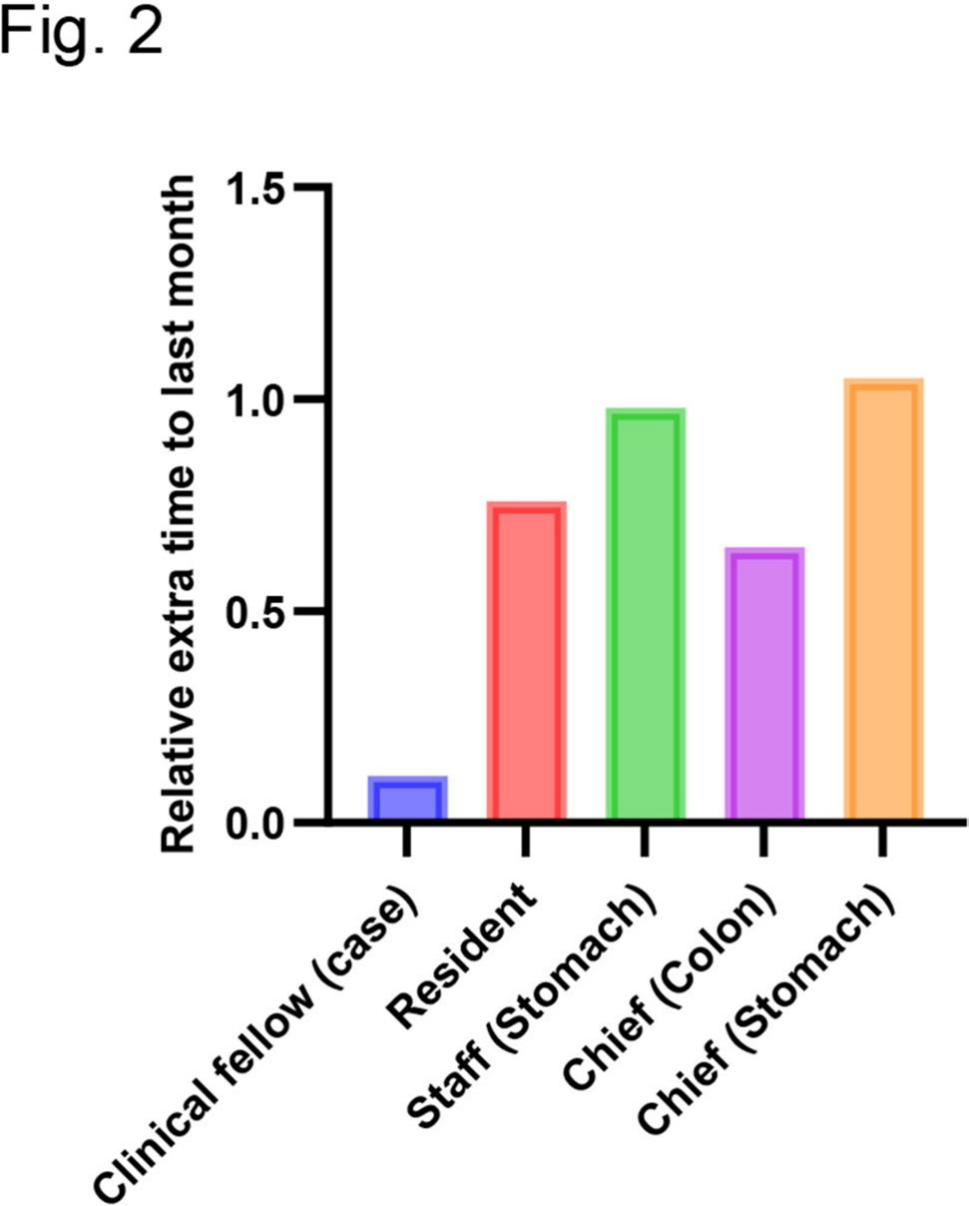
Impact on the other surgeons’ burden during his paternity participation time. Relative ratio of team members’ extra-time working (vs. previous month at delivery)

In Japan, female surgeons who choose to have a child tend to have less surgical experience than male surgeons because of inadequately supported post-maternity leave [1]. Male surgeons being able to take paternity leave based on their work–life balance will reduce the difficulties experienced by female surgeons trying to balance their maternity leave and career plan. It is critical to share the importance of diversity of career paths with co-workers.

Even in the US, it is still challenging for male surgeons to have enough paternity leave for fear of stigma from co-workers [2, 3]. To gain further insights into the effects of task shifting on their team, the surgeon in this case distributed a questionnaire using closed questions, to team members, focusing on the impact of task shifts, communication, work–life balance, and suggestions for improvement. Figure 3 summarizes the results of this questionnaire. The team's experience with task shifting during the 1-month paternity leave period yielded positive results, with all members reporting an interest in participating in future care of their children. One resident responded that they felt more pressure to complete their colleague’s tasks although their extra working time did not increase. It is difficult for surgeons to change the clinical system immediately. This successful and sustainable paternity participation for a short period (1 month) would still be limited to major hospitals where clinical work optimization and smooth communication in a team could facilitate the time off to support the individual. Moreover, work style reforms for doctors, which include restrictions on excessive extra working time, came into effect from April 2024 in Japan. Our changes and modifications such as task shifts might help all surgeons to reduce their extra working hours and increase their time off. Conversely, on reviewing our workflow, we also recognize the extent of inefficiencies in our previous processes. For the next step, task shift from surgeons to nurses and employing clinical or research assistants might help surgeons to make more time. Ultimately, there is a pressing need to create an environment where both male and female surgeons, including residents, can take parental leave.

**Fig. 3:**
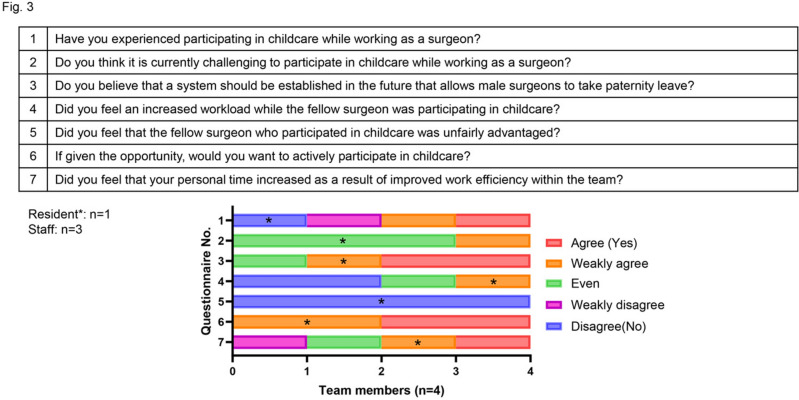
Questionnaire for clinical team members after the paternity participation time. Staff surgeons (*n* = 3) and Resident (*n* = 1). *: Resident’s answer

In summary, a long process is required to become an expert surgeon, but work–life balance is also very important. Therefore, sustainable paternity participation is a reasonable first step to become a “daddy surgeon” and attain balance between a life plan and a career plan. To achieve this, it is very important for a workplace to foster good communication so young surgeons can discuss their life plans with co-workers comfortably and supportively.
